# Supplementary material for: Are carbon emissions trading and green financial instruments synergistic? -Comprehensive quantitative research based on content analysis
Source: PLoS One. 2024 Mar 7;19(3):e0298601. doi: 10.1371/journal.pone.0298601 (PMC10919601; doi:10.1371/journal.pone.0298601)
Supplement: S1 Data — (ZIP) [file pone.0298601.s001.zip › Data/地级市Z_F工作报告.zip!/║■▒▒/╧σ╤⌠╩╨/└┤╫╘╣1⁄2╓┌║┼í╛┬φ┐╦╩2╛▌═°í┐_2013.docx]

各位代表：

　　现在，我代表市人民政府，向大会报告工作，请各位代表审议，并请政协委员和其他列席人员提出意见。

　　一、2012年工作回顾

　　2012年，在市委的坚强领导下，全市上下深入贯彻落实科学发展观，励精图治，夙兴夜寐，勤勉务实，扎实做好战略性、开创性、基础性、长远性工作，“两个中心”、“四个襄阳”建设取得了阶段性成果。

　　——经济逆势而进，主要指标在全省份额明显上升。预计全年实现地区生产总值2450亿元，同比增长12.5%；完成地方公共财政预算收入139.8亿元，同比增长43.5%。1至11月，固定资产投资、地方公共财政预算收入和地税收入分别增长43.2%、38.5%和50.5%，增幅均居全省市州第一位；规模以上工业增加值、固定资产投资、实际外商直接投资、外贸出口额和地方公共财政预算收入等5项指标，占全省比重比上年同期分别提高0.7、0.9、0.9、2.1和0.9个百分点。前3季度，在中部地区80个同类城市中，地区生产总值由上年的第10位上升到第5位。

　　——市场主体数量大幅增加，发展质量明显提升。市场主体增量工程成效显著。全年新增各类创业主体6.67万户，同比增长81.6%；新登记注册资本金303.6亿元，同比增长74.7%。市场主体规模加速壮大。新增规模以上工业企业168家，新增产销过亿元、过10亿元、过50亿元的骨干企业分别为100家、10家和3家，新增中国驰名商标10件；新增限额以上商贸企业251家，营业额过亿元的企业100家，其中过5亿元的企业达到10家。

　　——结构调整力度加大，抗风险能力明显增强。产业结构进一步优化。除汽车产业因宏观环境影响有所下降外，其它六大支柱产业均保持28%以上的增幅，新能源汽车产业成倍增长，新能源新材料产业成长为百亿产业。服务业结构明显改善，以云计算为主的高端服务业发展提速，现代服务业税收增速高于一般服务业28个百分点。投资结构明显优化。重大项目建设取得新的突破，开工建设5亿元以上投资项目259个，其中过10亿元的项目117个、过50亿元的项目8个，投资或产出规模过100亿元的项目12个。1至11月，工业投资力度持续加大，占固定资产投资的比重达到57.3%，同比增长37.9%；第三产业投资增势强劲，同比增长49.5%，大批文化旅游、商业、现代服务业项目开工建设，有力地拉动了固定资产投资和财政收入增长。

　　——新城新区框架拉开，未来发展空间明显扩大。以国际化视野推进城市东进。全力建设东津新区，5平方公里核心区房屋征迁和起步区拟征迁房屋评估锁定工作全面完成，100万平方米还建小区建设全面开工，34.6平方公里起步区“三纵五横”路网建设全面启动，内环东线、南线主干道贯通，大剧院等十大公共工程有序推进。以提升城市功能和品位为重点加快推进旧城更新。启动了主城区六大片区29个“两改”项目、8个城市综合体建设。以产城融合、城乡一体思维布局县（市）新城新区建设。六个县（市）新城新区实施各类建设项目551个，完成总投资403亿元，超过了过去5年的总和；征迁房屋总量达到591.59万平方米，是“十一五”时期的3.7倍。开工建设骨干路网134.5公里，建成84.8公里，覆盖面积157.7平方公里，拉开了新城新区框架；一批功能性项目陆续展开，特色产业园区建设加快推进，搭建了未来发展的新平台。

　　——政策体制效应充分显现，市场影响力和资源集聚力明显增强。“一主两副”政策优势和新的领导体制优势，极大地增强了襄阳集聚资源的能力，形成了科学发展、跨越发展的强劲气场。国家和省政策支持力度明显加大。汉江流域综合开发上升为国家战略，东津新区建设上升为省级战略，我市成为全国农产品现代流通综合试点城市、全国家政服务体系建设试点城市、国家级科技兴贸创新基地；争取中央和省项目总投资126.8亿元，同比增长22.2%。战略合作层次明显跃升。与17个国家部委、40家央企建立紧密联系，与清华大学等12所高校建立产学研合作联盟，校地企共建研究院（中心）7个。招商引资实现历史性突破。实收资本98亿元，实际到位资金806亿元，分别比上年增长118.7%和91.9%；新注册4个投资过100亿元、6个过50亿元的重大项目，英菲尼迪、中兴通讯等一批知名品牌和IBM、SK等9家世界500强落户襄阳。聚集资本要素的能力明显增强。新增各类金融机构36家，贷款余额突破1000亿元，比年初增加200多亿元；华海纤维在天交所成功上市。人才集聚效应明显扩大。深入实施“隆中人才支持计划”和“双千”专业人才引进计划，新引进7个海内外创新创业团队、618名博士硕士、855名各类专业人才。城市影响力明显上升。我市再次被《福布斯》评为“中国大陆最佳商业城市”，被国际知名投资机构仲量联行评为“中国新兴城市50强”。、

　　与此同时，国防后备力量建设不断加强，军政军民团结进一步巩固，统计、物价、民防、气象、档案、史志、民族宗教和对口支援西藏、新疆、恩施等各项工作都取得明显成效。

　　一年来，我们主要做了以下六个方面的工作：

　　（一）坚持顶层设计，科学谋划“两个中心、四个襄阳”建设。高起点编制规划。聘请一流规划团队，完成了“四个襄阳”发展战略规划、11个新区规划和34个专项规划，初步形成了比较完整的规划体系。系统谋划襄阳长远发展。制定工作路线图、时间表，将各项规划项目化、路径化。围绕规划培训干部。通过开设“四个襄阳”建设论坛，组织县（市）区和市直有关部门主要负责人到新加坡、深圳、昆山学习，举办科级干部规划读书班和县级干部十八大精神研修班，组织县（市）新区规划和产业规划专题演示汇报会，培养干部国际化视野和战略思维能力，用科学规划指导发展。汇民智、聚民力，完善规划、落实规划，使“两个中心、四个襄阳”建设成为全市人民的广泛共识和共同行动。

　　（二）坚持综合施策，全力化解经济下行压力。我们始终与企业同舟共济、共度时艰，以“组合拳”营造稳增长的“小气候”。以做大做强投资拉动经济增长。策划实施一大批重大产业项目和重大基础设施建设项目，强化了投资支撑。以推动工业跨越发展促进经济增长。设立15亿元产业发展基金，支持企业重组扩张、搬迁改造、新上项目，出台政策措施推动优势产业集聚集群集约发展。以推进全民创业激活经济增长。出台支持全民创业政策18条、回归创业政策42条、微型企业发展政策20条和民营经济发展政策42条，我市成为全省全民创业门槛最低、创业活力最强的城市。以帮扶企业解困稳定经济增长。采取短期无息借款、贷款贴息、担保贷款补助等17条政策措施，帮助困难企业摆脱困境，支持南漳、保康、谷城三县受灾工业企业恢复生产。

　　（三）坚持改革创新，培育经济跨越的比较竞争优势。系统推进组织经济工作专业化改革，完善“六个一”产业发展推进机制；坚持把优质资源匹配给优秀企业，对未来五年产出规模有望过100亿元、50亿元和10亿元的109家骨干企业，分类召开现场办公会，量身定做扶持政策。创新招商引资和重大项目建设推进机制，大力推进专业招商、产业链招商，积极拓展股权招商、科技招商，千方百计引进培育战略性资源；实行重大项目集中开工制、服务秘书制、督导协调制、媒体公示制。创新资本集聚机制，在全省率先运用财政杠杆激活金融资本，建立了信贷风险补偿机制，出台鼓励金融机构和股权投资机构投资产业发展政策，建立财政专项资金跟投信贷和股权投资项目机制。加强科技创新能力建设，实施128个国家和省科技项目，新增43家高新技术企业、8家省级创新型企业，宇清传动插电式商用车自动变速驱动系统项目列入国家“863”计划，东风股份新能源汽车产业技术开发项目列为工信部技术创新工程。深入开展“管理创新年”活动，推进政府工作流程、行政审批、市场监管、公共服务等九大创新，成为全国行政审批事项最少的城市，建立行政审批许可和超时默认制度，在全国首创行政审批联审联办网络系统，在全省率先对涉企行政执法检查和处罚行为实行全时段、全过程电子监察，设立了“12345”公共服务热线。构筑大开放平台，建设“大通关”体系，启动了襄阳机场改扩建工程，新开通航线6条，进出港旅客同比翻一番；开通了襄阳至宁波铁海国际联运通道；规划建设国际化产业园区；新增7个国际友好城市。

　　（四）坚持城镇化引领，加快城乡一体化进程。着力推进城市功能设施建设。主城区实施了307个总投资380亿元的功能性项目。24条城市道路改造竣工通车，唐白河大桥主体工程完工，汉江卧龙大桥、东津大桥等重点工程加快推进；城市综合环境提升工程深入推进，硬化绿化亮化背街小巷40条，改造人行道17.6万平方米，启动建设南渠生态廊道和岘山森林公园等生态景观项目11个，汉江亮化一期工程竣工，新增绿地面积12.8万平方米，主城区空气质量良好天数保持在310天以上，汉江干流襄阳段水质保持国家二类标准。深入推进“五城同创”，综合治理市区交通拥堵初见成效，我市被评为“湖北省森林城市”。以战略眼光推进交通枢纽建设。4条高速公路、9条国省公路绕城改线工程和7个大型物流园区建设全面展开，襄阳火车东站投入运营，交通建设总投资是上年的3.4倍。城乡一体化发展步伐加快，市级重点推进襄州和襄城两个城乡一体化综合试点，每个县（市）区实施一个整镇推进试点；现代农业强市建设扎实推进，新增1家国家级、19家省级农业产业化龙头企业；新流转97.2万亩农村土地，新增840个农民专业合作社；完成5大灌区节水改造和140座小型水库除险加固，整治16595口堰塘，新打858口抗旱机井，农业综合生产能力明显提升，成为长江流域首个粮食产量过百亿斤的城市。

　　（五）坚持文化立市，加快打造国内外有重要影响的文化旅游名城。实施文化襄阳建设三年行动计划，推进国家公共文化服务体系建设示范区和文化产业发展示范区创建工作。全面启动了主城区“四馆一院”（博物馆、图书馆、群艺馆、美术馆、大剧院）和县市“两馆”（文化馆、图书馆）建设，农家书屋、乡镇文化站、信息资源共享工程、广播电视村村通等文化设施实现全覆盖。争当“襄阳百杰”、“书香溢襄阳”全民阅读等群众文化活动深入开展，“群星音乐厅”、“襄阳公共文化探索与研究”两个文化品牌获得文化部“科技创新奖”。我市被评为“全国文化体制改革工作先进地区”。全面推进“四城（襄阳古城、大唐影视城、汉城、三国文化城）四园（米芾文化创意产业园、文化传媒产业园、汽车文化产业园、文化科技产业园）四区（三国文化旅游区、大荆山生态旅游区、都市文化休闲旅游区、汉江风光带旅游区）”建设，大力实施古隆中景区改扩建、唐城影视城等23个重大文化旅游产业项目，完成投资78.5亿元。全年接待游客2350万人次，实现旅游总收入150亿元，同比分别增长30%和27%。成功举办了一届高层次、国际化、群众性、襄阳味的诸葛亮文化旅游节，“隆中对·创新与人才”活动取得丰硕成果，被评为“中国节会十大品牌”，襄阳的国际知名度明显提升。

　　（六）坚持民生优先，提升市民的幸福感和满意度。关系民生的“十件实事”基本完成。全市新增就业11.3万人，城镇登记失业率控制在3.78%。预计城镇居民可支配收入和农民人均纯收入分别提高1996元和996元，增长13%和13.2%。城镇职工“五险”扩面新增26万人次，城乡居民养老保险制度实现全覆盖，城镇职工养老金标准月均提高233元，城乡低保月补助标准分别提高30元和25元，城镇职工、居民医保报销限额和新农合住院补偿限额均高于省定标准。残疾人生存和发展状况明显改善。3万贫困人口摆脱贫困，扶贫工作在全省考核中名列第一。改造农村危房1.7万户，新建普通商品住房47300套、各类保障性住房13992套，城镇居民人均住房面积由28.2平方米提高到30平方米。21.9万农村人口的饮水不安全问题得到解决。17.9万受灾群众得到妥善安置。城乡学前教育、义务教育和特殊教育条件进一步改善，31158名进城务工人员子女和72639名留守儿童的上学问题得到解决；湖北文理学院创建综合性大学步伐加快、理工学院新校区建设进展顺利，襄阳汽车职业技术学院新校区建成投入使用，三大职教联盟组建基本完成。医药卫生体制改革进一步深化，基层卫生服务供给能力不断增强。体育、人口和计划生育等工作取得新的成绩。切实关爱特殊人群，新建200个留守儿童服务站，组织5100名爱心妈妈结对帮助留守儿童6700人。应急管理和安全生产、食品药品安全监管持续强化，安全生产指标控制在省定目标以内。社会管理创新走在全省前列，“一站四化”的社区公共服务体系进一步完善，信访法治化体系初步建立；推动警务改革，建立移动警务平台，加强了社会面控制，刑事案件发案率下降9.89%，群众安全感指数和治安满意度分别比上年提高5.4和3.6个百分点。

　　一年来，我们在履行职责的过程中，高度重视政府自身建设，完善工作规则，规范决策程序，促进科学决策；出台政府投资工程管理办法、政府投资项目审计监督办法、BT项目管理办法等，对政府投资工程实行全程跟踪审计。开展“市民问政”，强化“治庸问责”，有力促进了政风行风好转。坚持重大事项向人大报告和政协通报制度，认真办理人大代表议案、建议和政协建议案、提案，自觉接受人大、政协监督和社会监督。法治城市创建扎实推进，依法行政水平进一步提高。认真落实廉政建设责任制，有效遏制了腐败案件的发生。

　　各位代表，过去的一年极不平凡，取得的成绩来之不易。这是省委、省政府正确领导、倾力支持的结果，是市委统筹全局、科学组织的结果，是全市人民团结奋进、真抓实干的结果。在此，我代表市人民政府，向全市人民，向所有纳税人，向各位人大代表、政协委员，向各民主党派、工商联、人民团体和无党派人士，向驻襄人民解放军、武警官兵和公安干警，向所有关心支持襄阳发展的同志们、朋友们，表示衷心的感谢，致以崇高的敬意！

　　在总结成绩的同时，我们清醒地看到发展中还存在不少矛盾和问题，主要表现在：经济发展外部压力持续加大，土地、资金等生产要素和环境资源约束日益趋紧；产业结构不优，工业主导产业相对单一的问题仍然突出，现代服务业发展滞后，面临经济增长与产业转型双重压力；中心城市能级与建设“两个中心”的要求不相适应，城镇化水平和质量不高，统筹城乡发展任重道远；社会事业发展相对滞后，经济社会转型中的矛盾持续显现，社会建设和管理任务加重；科学发展的体制机制有待完善，改革的系统性、协调性和针对性还需增强；政府行政效能和政府工作人员专业素养还需要进一步提升；等等。对此，我们将采取积极有效措施，切实加以解决。

　　二、2013年工作部署

　　2013年，是全面贯彻落实党的十八大精神的开局之年，是加快建设“两个中心、四个襄阳”的关键之年。当前，我们面临的外部环境仍然复杂多变，世界经济的各种风险和不确定因素依然很多，我国经济企稳回升的基础尚不稳固，外需市场萎缩，内需市场不旺，区域经济竞争更加激烈，给我市经济增长带来了很大压力。同时我们也要看到，国内经济总体向好的基本面没有改变，我市仍处于加快发展的重要战略机遇期。今年国家继续实施积极的财政政策和稳健的货币政策，加大对“三农”、基础设施、民生等领域的投入，保持社会融资规模合理增长，只要我们顺应国家宏观政策，争取实施一批符合国家政策支持和资金投向的项目，就一定能够保持足够的投资强度；我国新型工业化、信息化、城镇化和农业现代化加速推进，蕴含着巨大的内需潜力和市场空间。我市工业基础较好，农业比较优势明显，城镇化空间很大，只要我们坚持以城镇化为引领，持续推动改革创新，就一定能够增创新的比较竞争优势；国家将加快实施振兴老工业基地和汉江流域综合开发战略，省委、省政府不断加大支持“一主两副”中心城市发展的力度，为我市提供了重大发展机遇，只要我们抓住机遇、善于谋划，就一定能够在更高平台、更大范围配置资源；“两个中心、四个襄阳”规划全面完成，为当前和今后一个时期的发展打下了良好基础；近年来，我们推出的一系列改革创新举措，所做的战略性、开创性、基础性和长远性工作，必将对“两个中心、四个襄阳”建设产生历史性的影响。只要我们保持这种抢前争先的劲头、攻坚克难的勇气、苦干实干的作风，就一定能够在建设“两个中心、四个襄阳”的征程中创造新的业绩。

　　今年政府工作的总体要求是：以邓小平理论、“三个代表”重要思想、科学发展观为指导，深入贯彻落实党的十八大精神，围绕建设“两个中心、四个襄阳”的战略目标，坚持以做大总量提高质量为首要任务，着力推进经济转型跨越；以城镇化为引领，着力推动“四化”同步发展；以改革创新为动力，着力培育经济跨越的比较竞争优势；以保障和改善民生为落脚点，着力加强社会建设，为率先全面建成小康社会奠定坚实基础。

　　经济社会发展的预期目标是：地区生产总值增长13%，规模以上工业增加值增长18%，全社会固定资产投资增长40%，社会消费品零售总额增长16%，实际外商直接投资和出口额分别增长15%，地方公共财政预算收入增长22%，城乡居民人均可支配收入分别增长13%；城镇登记失业率控制在4.5%以内。全面完成省政府下达的物价调控、节能减排、计划生育和安全生产等约束性指标。

　　围绕实现上述目标，重点做好七个方面的工作：

　　（一）着力做大做实做强投资

　　坚持把扩大投资作为推进结构调整的关键举措，以项目建设支撑转型跨越。

　　强力推进招商引资。整合招商资源，完善激励、考核、服务等推进机制，建立重大招商项目评估机制。进一步创新招商方式，深入实施产业链招商行动计划，向境内外重点地区派驻专业招商团队，紧盯国际国内产业龙头企业和行业领军企业，突出战略性新兴产业、先进制造业、生产性服务业等领域的招商，巩固股权招商、科技招商，突破产业集群招商和稀缺资源招商，吸引世界500强、中国100强在襄阳设立研发、销售、结算中心和地区总部。力争全年新注册投资过100亿元的项目2-3个、过50亿元的项目5-6个、过10亿元的项目50个，全年招商引资到位资金突破1000亿元。

　　加快重点项目建设。全力抓好182个投资过5亿元的省市重点项目建设，确保全年新开工投资过10亿元的项目50个、过50亿元的项目10个，开工投资过100亿元或产出规模过100亿元的项目12个。重点抓好东风乘用车零部件产业园、襄阳磷化工循环经济产业园、谷城再生资源利用产业园、珠江数控装备产业园、阳光凯迪低碳经济产业园等一批重大产业项目建设，全力促进北煤南运铁路通道、襄阳新港、新集水电站、国省干线公路改造等一批重大功能性、支撑性项目开工，推动武襄动车运营公交化。扎实做好郑万高铁、武襄城际铁路前期工作，力争大唐热电联产、华润电力和安能热电燃气机组等能源项目获得核准批复。抢抓国家继续实施积极财政政策的重大机遇，策划申报一批基础性、支撑性、功能性、公益性的重大项目。

　　全面激活民间投资。全面落实促进民营经济发展和扩大民间投资的政策措施。进一步拓宽民间投资领域，降低准入门槛，创造公平竞争、平等准入的市场环境。大力实施市场主体增量工程、民营企业成长工程和民营经济提质工程，支持民营企业引进人才、技术创新、技改扩能、创建品牌、开拓市场，支持民营企业通过上市、发行债券、引进股权投资等多渠道融资，促进民营经济壮大总量、提升质量，带动民间投资规模快速扩张。

　　（二）着力推动产业转型升级

　　坚持做大总量与调整结构并重，大力推进产业转型升级，加快形成具有比较竞争优势的现代产业体系。

　　强力推进工业转型跨越。深入实施工业跨越式发展行动计划。突破性发展战略性新兴产业。以数控机床、轨道交通、航空航天、节能环保等装备制造产业为重点，加快打造4个百亿级高端装备制造产业基地；以开发利用生物质能源和页岩气，突破性发展特种化学材料、光学材料和电子材料产业为重点，加快打造全国有重要影响力的新能源新材料产业基地；以培育云计算、北斗导航、汽车电子等产业为重点，加快打造全国重要的新一代信息技术产业基地；以东风新能源汽车阵地为龙头，加快新能源整车基地建设，引导关键零部件及材料产业集聚，促进新能源汽车产业化、规模化发展；充分发挥谷城国家级“城市矿产”示范基地的国牌效应，着力培育再生铅、再生铝、再生钢等循环经济产业链，加快打造千亿级资源再生利用产业。大力推进传统产业升级。加快实施以东风公司为主体的“四个百万”工程，加快建设东风特种车基地，引导关键零部件集聚发展，促进汽车零部件产业完善链条、做大规模、提升层次；引导纺织服装、建材冶金、医药化工等传统优势产业向研发设计、品牌营销、终端产品等高端环节延伸；加快推进信息化与工业化深度融合，促进全产业链整体升级。大力发展矿业经济。聘请专业团队找矿、探矿，高水平编制矿产资源综合开发利用规划，大力引进全球矿产资源综合开发利用龙头企业，整合利用、高效开发我市矿产资源。大力推动产业集聚集群集约发展。按照产城融合的要求，加快建设主城区“两区十园”和县（市）特色产业园区，推动产业链垂直整合。大力实施骨干企业成长工程。力争全年新增4家产值过50亿元、13家过10亿元、130家过亿元的骨干企业，争创3-5个中国驰名商标。着力打造公共服务平台。力争建成国家动力电池产品质量监督检验中心、国家汽车零部件检测重点实验室、区域性食品药品检验检测中心，争创国家级工业产品质量控制和技术评价实验室，加快建设襄阳科技城、“云制造”基地和市中小企业综合服务中心。

　　突破性发展现代服务业。加快推进城市商圈建设。高起点编制商圈规划，启动一批商圈建设项目。加快现代市场体系建设。启动建设主城区10平方公里综合交易市场集聚区，加快建设国际商贸城和华中工业博览城，完善新农村现代流通网络。突破性发展现代物流业。加快推进余家湖煤炭储备基地、襄阳北国际物流园、襄阳西现代物流园、襄阳物流信息中心等重大物流项目建设，争创5家国家A级物流企业。大力发展信息服务业。着力打造“襄阳云谷”，加快建设中国移动湖北公司襄阳创新基地“四个中心”、中部呼叫产业城、IBM华中云计算中心、联通公司鄂西北通讯枢纽中心等项目，建设中部汽车信息服务外包基地、全国汽车零部件电子商务平台，争创国家级新一代信息技术应用示范基地。

　　加快发展现代农业。大力推进农产品加工业提档升级。加快实施正大100万头生猪产业化项目、倪氏国际玫瑰产业园、中兴农产品加工产业园等14个省市级重点项目，新增1个产销过100亿元、2个过50亿元的骨干企业，争创3-5个“中国地理标志”农产品。大力推进农业规模化经营。完善农业科技推广体系，集成推广优质良种和先进耕作技术，集中连片、整市推进粮食高产示范创建，巩固百亿斤粮食大市地位；加快汉江流域高效农业带综合开发，新建100万亩特色农产品生产基地；扩大畜牧业规模化养殖，新建国家、省、市级畜牧示范养殖场5个、10个、150个；加快襄州现代农业综合示范区建设步伐，启动樊城都市农业示范区建设。加强农业基础设施建设。加快推进灌区节水改造、大型泵站更新改造、小农水重点县项目建设和小（二）型水库除险加固工程；抓紧推进鄂北水资源配置工程前期工作，完成汉江东津段、南河、蛮河治理和引丹入襄工程冢子湾水库除险加固；积极推进农村土地整治，大规模建设高标准基本农田。深化农村改革。坚持和完善农村基本经营制度，全面开展农村土地确权登记工作；完善农村土地流转机制，新流转180万亩；大力培育新型农村合作组织，实现全市行政村农民专业合作社全覆盖；深入推进集体林权制度改革，深化城中村、城郊村、园中村集体资产产权制度改革，实施农业水价、水管体制和小农水产权制度改革，增强农业农村发展活力。

　　（三）着力加快城镇化进程

　　全面推进新城新区建设。举全市之力建设东津新区，实现5平方公里核心区基础设施全覆盖，内环线东津段和东津大桥建成通车，“三纵五横”道路主干道贯通；加快建设市民中心等“十大公共工程”；加快建设还建小区，确保50万平方米安置房投入使用、150万平方米保障房和安置房主体完工、100万平方米安置房完成工程量的50%。大力推进县（市）新城新区建设，全面建成起步区骨干路网体系，推动一批公共工程开工，力争河谷新城和枣阳两个副中心、襄南宜一体化和保康“都市襄阳后花园”建设取得重大进展。

　　加快推进旧城更新。按照“征转分离、统征统迁、集中安置、社区融合”的原则，加快推进城市“两改”，扎实抓好主城区29个“两改”项目建设，完成征迁48712户、905万平方米，建成安置房567万平方米，开工建设新襄阳客厅等13个城市综合体，在每个城区抓好1-2个老旧小区改造试点；着力提高居民小区物业服务水平，新建和老旧小区物业服务覆盖面分别达到100%和50%。推进城市“两迁”，实施五年行动计划，加快内环线以内的工业企业和专业市场外迁步伐。

　　加快推进城市功能开发。实施城市道路畅通工程，强力推进主城区“两轴、三环、九放射”骨干路网建设，确保南北轴线全线贯通，东西轴线大庆路至东外环全线开工，内环线建成通车，中环线完成60%的工程量，外环东线全线开工，四条出口路建成通车，南湖宾馆至襄阳机场景观大道和鱼梁洲环岛景观路建设全面启动，新建、改建城市道路286公里。大力发展公共交通，提高市民出行便利度。加强市政基础设施建设，重点实施主城区六大水系治理工程、十个污染治理工程、八个泵站配套工程、七个排水能力提升工程和三大综合景观工程，新建和改造排水管网420公里，建成供水管网95公里、燃气管网55公里、加气站7座。

　　大力推进中心镇和新型农村社区建设。推进市域城镇体系规划管理全覆盖。加快推进12个中心镇建设镇级市，完成中心镇新区规划，加强基础设施建设，提升承载功能。加快推进襄城尹集城乡一体化试点建设；按照一个县（市）区抓好一个整镇推进试点、一个乡镇抓好一个整村推进试点的要求，加快建设新型农村社区；深入开展“万名干部进万村洁万家”活动，加快建设天蓝地绿水净的美丽乡村。新建120公里县乡等级公路和600公里通村水泥路，进一步改善农村交通条件。深化户籍制度改革，有序推进农业转移人口市民化。

　　深入推进“五城同创”。力争国家森林城市创建通过验收，全国文明城市、国家卫生城市、国家生态园林城市和国家环保模范城市创建工作取得积极进展。深化“城管革命”，加大市容专项整治力度，深入开展“环境美好示范路”和“环境美好示范小区”创建活动，建成数字化城市管理系统。

　　（四）着力打造文化旅游名城

　　坚持文化立市战略，大力实施文化襄阳建设三年行动计划，加快创建国家级公共文化服务体系示范区和国家级文化产业示范区，增强文化软实力，提升城市竞争力。

　　着力优化人文环境。坚持以社会主义核心价值观为统领，深入推进群众性精神文明创建，广泛开展争创“襄阳百杰”、学雷锋志愿服务、“书香溢襄阳”全民阅读等群众文化活动，加强对市民法治意识的培养和现代文明教育，大力普及科学知识、提高全民科学素养，引导广大市民牢固树立“精进致远、包容大气、创新创造、法治诚信、勤勉务实”的人文理念；建立“市民义务劳动日”制度，培养广大市民对城市发展的责任感。以文化渗透促进市民现代文明素养的提升，努力打造科学发展、跨越发展的“精神高地”。

　　大力发展公共文化事业。加强公共文化设施建设。完成市博物馆、大剧院主体工程；加快市县两级公共图书馆、文化馆数字化改造，开展乡镇文化综合体建设试点；推进城乡有线电视数字化改造，新增2万户农村数字电视用户，实现主城区数字电视双向网络全覆盖。积极创建全国残疾人文化建设示范市。加强文化精品创作生产和历史文化遗产保护利用，抓好商业会馆修缮，推进一批古镇古村、古街古巷的保护和开发；整理非物质文化遗产，进一步做好襄阳古城墙申遗工作。

　　突破性发展文化旅游产业。大力推进主城区“四城四园四区”建设。建成大唐影视城，复建襄阳古城城门楼，加快建设汉城，启动建设三国文化城；全面推进米芾文化创意产业园、文化传媒产业园、汽车文化产业园和文化科技产业园建设；启动建设古隆中新门景区、核心景区和演艺区，加快建设岘山景区、以鱼梁洲为龙头的汉江风光带，确保习家池核心区、鹿门山核心区、薤山核心区、九天玄女旅游区、保康九路寨等项目建成运营。完善旅游服务体系，建设襄阳旅游接待中心；高水平策划推进旅游营销，继续办好诸葛亮文化旅游节，提高襄阳文化旅游品牌知名度。

　　（五）着力加强生态文明建设

　　大力推进资源集约节约利用。坚决执行最严格的耕地和水资源保护制度。探索建立产出贡献与用地指标挂钩的激励机制，鼓励集约节约高效用地；强力推进城乡建设用地增减挂钩、低丘缓坡荒滩未利用地开发和“三旧”改造试点，拓展土地利用空间。强力推进节能减排，扎实推进低碳城市试点，提高新能源和可再生能源利用比重，全面完成国家节能与新能源汽车示范推广试点城市的示范任务。加强气象、地质、地震等灾害防御体系建设。

　　加强自然生态系统和环境保护。实施生态修复工程，大力开展植树造林，加快构建“一城两带三网四区七廊”为主的生态系统。实施“蓝天碧水”工程，加大城市空气污染治理力度；加强“一江五河”等重点流域污染防治，严格落实汉江保护责任制，强化汉江流域水环境综合治理，加快建设沿江城镇污水处理设施，确保汉江干流襄阳段水质稳定在现有水平，促进主要支流水质进一步改善。深入推进生态县、生态镇、生态村创建工作，探索建立城乡生活垃圾一体化处理机制，加强农村面源污染和畜禽养殖污染治理。

　　加强生态文明制度建设。探索建立体现生态文明要求的目标体系、考核办法和奖惩机制。建立健全清洁生产机制，进一步加强对化工、冶炼、涉重（重金属）等重点行业、企业的环境监管。健全生态环境保护责任追究制度和环境损害赔偿制度。加强生态文明宣传教育，增强全民节约意识、环保意识、生态意识。

　　（六）着力推进改革开放创新

　　大力推进各项改革。深化行政体制改革。深入推进管理创新，实施“效能新政”，构建行政审批项目动态管理机制，建立网上政务大厅，推进服务流程互动，加快打造国内行政审批“事项最少、流程最优、收费最低、监管最好、效率最高”的城市。稳步推进“大部门”改革，积极推进“大科室制”改革试点。大胆探索公务员聘任制和政府雇员制。推进高新区、经开区管理体制改革，抓好县（市）区和乡镇行政管理体制改革试点。全面推进事业单位分类改革。深化组织经济工作专业化改革，建立产业集聚集群集约发展的专业化推进机制，建立整体联动、专业化运作的招商机制。加快推进城市公用事业体制机制改革。扎实做好第三次全国经济普查和投入产出调查工作。

　　大力推进科技创新。深入推进国家创新型城市创建工作。加强科技创新载体建设，加快引进跨国公司、知名高校、科研院所在襄阳设立研发中心或分中心，加快建设7个校地企研究院（中心），创建一批国家和省级重点实验室、工程研究中心、企业技术中心、校企共建研发中心。加快推进高新区国家级创新型特色园区建设，推动高新区创建国家科技与金融结合试点园区，建立区域性科技创新服务中心。加快培育创新型企业，深入实施科技型中小企业成长路线图计划，重点推进130个产学研合作项目和100个科技创新项目。深入实施“隆中人才支持计划”、“产业创新人才培养计划”和“科技人才创业计划”，着力在引进海内外高层次人才、培育产业创新团队和科技领军人才上实现新突破。

　　大力推进金融创新。加快建设区域性金融中心，力争在推动金融机构入驻东津新区设立地区总部和后台中心上取得实质性进展。完善金融组织体系，引进各类金融机构8家以上，积极筹建地方法人银行；力争在引进和培育投资公司、融资租赁公司、消费金融公司和财务公司上取得突破。完善信贷激励和风险补偿机制，鼓励银行业金融机构利用银行间市场为企业发行多种直接融资产品，加快融资担保体系建设，全年新增社会融资总额450亿元。力争30家上市后备企业与股权投资机构成功对接，2至3家企业上市；争取高新区获得“新三板”扩容试点资格，推动一批企业在武汉股权托管市场挂牌。加快建立要素交易市场，规划建设全国汽车交易中心和农产品交易中心。完善市建投集团“1+N”体制机制，增强投融资能力。优化金融生态环境，加强金融机构监管，维护金融稳定。

　　大力推进城市国际化。制定实施襄阳城市国际化规划。加快建设国际产业园、国际化社区，创办国际学校；着力构筑“大通关”体系，大力推进襄阳机场改扩建工程，加快申报一类航空口岸，启动建设电子口岸信息平台，加快建设集装箱办理站；大力建设“无水港”，推进铁海联运；争取与渝新欧、汉新欧等大陆桥的重要节点连接贯通，探索国际铁路联运，启动综合保税区建设。争创国家级家纺和汽车零部件出口基地、省级农产品和服务外包基地；坚持引资与引技、引智、引制（先进制度）相结合，提高利用外资质量和效益。大力实施企业“走出去”战略，支持一批优势企业充分利用全球资源和市场。加强与国际友好城市的交流合作，大力推进城市科教文化国际化建设，加快建设高新区国家级国际科技合作基地，争取与国际知名企业共建战略性新兴产业技术联盟。

　　（七）着力在改善民生和创新管理中加强社会建设

　　坚持民生优先，切实解决好人民最关心最直接最现实的利益问题，努力让全市人民过上更好的生活。

　　努力提高居民收入。实施居民收入倍增计划。大力推进创业富民，加大国家创业型城市创建力度，全面落实全民创业扶持政策，完善创业服务体系，支持全民创业和小微型企业发展。大力推进就业惠民，完善就业援助机制，建立面向全体劳动者的职业培训制度，实行城乡统一的就业补贴政策，积极创建全国职业培训示范城市。大力促进企业职工增收，建立最低工资标准动态调整机制，探索建立企业增薪激励机制，全面推行工资集体协商制度。大力促进农民增收，全面落实国家强农惠农富农政策，深入实施科技入户工程，鼓励开展土地股份合作，完善产业化龙头企业与合作组织、生产基地和农户的利益联结机制，加快发展劳务经济。大力开展低收入居民援助，建立逐步提高社会救助标准、城乡居民最低生活保障标准和老年居民各类补助标准的长效机制，完善社会救助和保障标准与物价上涨挂钩联动机制，确保低收入群体生活逐步改善。

　　办好人民满意的教育。继续实施学前教育三年行动计划，力争学前三年入园率达到70%以上。确保所有县（市）区义务教育均衡发展通过省级验收。扩大优质高中规模。保障进城务工人员子女公平接受义务教育，让更多的农村学生享受省级示范高中优质教育，启动建设市特殊教育中学。全力支持湖北文理学院创建综合性大学，支持襄阳职业技术学院创建国家骨干重点高职院校，加快襄阳技师学院东津新校区建设。

　　统筹推进城乡社会保障体系建设。完善社会保障制度，实现城乡居民养老保险制度全覆盖；继续推进社保扩面，稳步提高保障待遇。加快建设保障性住房和中小户型、中低价位普通商品住房，推进住房公积金制度全覆盖，完善住房保障体系；加强房地产市场调控，保持平稳健康发展。完善社会救助体系，全面推行城乡困难群众医疗救助即时结算，积极探索重特大疾病救助办法。大力发展慈善事业、红十字事业，加大扶老、救孤、助残力度，继续抓好农村福利院改扩建，加快建设市儿童福利院二期工程和市残疾人康复技术资源中心。强力推进南河流域为重点的扶贫开发，确保5万人脱贫。

　　提高人民健康水平。加快建设区域性医疗中心，启动建设公共卫生中心。深入推进医药卫生体制改革，加强基层卫生、疾病预防控制和卫生应急服务体系建设，深入开展“健康襄阳全民行动”和爱国卫生运动，力争基本医疗卫生服务的公平性、可及性居全省前列。大力发展体育事业。全面推进免费孕前优生健康检查，提高出生人口素质；稳定低生育水平， 综合治理出生人口性别比，促进人口均衡发展。完善食品药品安全监管机制，切实保障人民群众身体健康和生命安全。

　　加强和创新社会管理。完善“一站四化”社区公共服务体系，大力发展社区服务性社会组织，加快推进公共服务进社区。以创建法治城市为抓手，加快建立社会管理法治化体系。健全社会矛盾大调解体系，完善信访工作法治化体系；把依法治理作为社会管理的关键措施，不断提高各级政府依法行政的意识和能力，提高执法部门公正执法的水平和权威，提高社会依法解决矛盾纠纷的便利度和自觉性；加强平安建设，深化警务改革，健全移动警务机制和网格化巡逻机制，完善社区警务机制，构建立体化社会治安防控体系，严厉打击违法犯罪行为，保障人民群众生命和财产安全。以创建本质安全型城市为抓手，强化公共安全体系和企业安全生产基础建设，防止重特大安全事故发生。

　　在做好上述工作的同时，认真办好关系民生的十件实事。

　　1、大力推进全民创业就业。全面实施普惠的就业技能提升计划，培训劳动力12万人，新增城镇就业10万人，转移农村劳动力6万人，再就业2.6万人，支持1.75万人创业，发放小额担保贷款3.5亿元以上，力争全市全年新增微型企业5000家以上。

　　2、推进城市标准化社区服务中心建设。全市建设标准化社区服务中心100家，其中主城区40家、县（市）60家。

　　3、推动基本公共服务体系提档升级。完成主城区6家社区卫生服务中心标准化建设，改造升级20家菜市场，新增30家社会保险交费网点。

　　4、完善特殊群体救助政策。新建留守儿童爱心服务站200所；为1万名特困残疾人发放生活补助，为1000名贫困残疾人配置辅助器具；改造农村危房8000户，实行扶贫搬迁800户；实施“民生一元保险计划”，为因见义勇为和自然灾害导致伤残或者死亡的居民提供保险保障。

　　5、大力发展公共交通。制定实施公共交通发展规划，更新城市公交客运大巴200辆，新开公交线路2条，新增里程100公里，建设一条快速公交示范线路，改造建设公交港湾式站台20个，基本实现主城区公交线路全覆盖。

　　6、实施蓝天碧水工程。开展城乡生活垃圾一体化处理试点工作，在农村探索建立“村收集、镇转运、县处理”的垃圾清运处理体系，在市区试行生活垃圾分类处理；新增城市绿地100万平方米，新建公园4个、游园6个，建设绿道110公里；再解决20万农村人口饮水安全问题。

　　7、加大教育投入。全市新建幼儿园10所、改建幼儿园30所；完成200所义务教育学校标准化建设。

　　8、实施文化惠民工程。送戏下乡演出1000场以上，送电影下乡2.5万场次以上；开展“欢乐襄阳”文化惠民活动150场，“群星音乐厅”活动12场以上，好戏大家看·优秀剧目展演活动12场；新建社区流动图书站30所。

　　9、加大保障性住房建设力度。开工建设各类保障性住房（含人才公寓）15000套，竣工9000套；建设保障房住宅小区物业管理规范化试点10个。

　　10、大力发展社会化养老服务。全市新增养老床位1000张；启动襄阳市社会养老服务中心建设项目，开工建设市级老年公寓和樊城区社会福利院。

　　三、加强政府自身建设

　　面对“两个中心、四个襄阳”建设的历史使命，面对人民对美好生活的新向往，我们深感责任重大，不敢有丝毫懈怠。新的一年，我们将以规划落地、项目落地为重点，深入开展“落实年”活动，扎实推进法治政府和服务型政府建设，努力把襄阳建成行政效能高、社会秩序好、发展环境优的城市。

　　（一）强化责任担当。落实源于责任，责任重于泰山。全体公务员要坚持原则、敢于负责、勇于担当。面对“两个中心、四个襄阳”建设的重任勇于担责，潜心静气，凝神聚力，坚持做打基础、利长远的工作，一步一个脚印地去奋斗，一点一滴地去积累；面对发展中的各种矛盾敢于担难，以敢于碰硬、敢打胜仗的勇气奋发作为，以知难而进、锲而不舍的奋斗精神攻坚克难；面对前进中的各种挑战敢于担险，凡是对襄阳长远发展有利的事，凡是符合绝大多数群众根本利益的事，认准了就不怕遭非议、不怕受指责、不怕出失误，敢闯敢冒、敢为人先，义无反顾、勇往直前往前干。

　　（二）提升能力素质。能力素质决定落实成效，落实成效检验工作水平。全市各级公务员都要把加强学习调研、提高专业素质作为胜任岗位、履行职责的第一需要，坚持在学习中提高专业素质，以学习自觉促进行动自觉，增强推动工作落实的“正能量”。各级政府和部门的领导干部要通过坚持不懈的学习，不断提升把握和运用市场经济规律、自然规律、社会发展规律的能力，不断提升科学决策、民主决策的能力，不断提升全球思维、战略思维能力，不断提升驾驭复杂局面、系统思考问题和解决问题的能力。突破传统的思维习惯，敢于创新，善于创造，以创新的举措破解发展难题，突破瓶颈制约。

　　（三）创新工作机制。建立运转协调的落实机制，实现规划设计、责任分工、系统推进、动态评估和信息反馈等环节的无缝对接。推行政府绩效管理，健全重点工作全程考核推进机制，将重点工作规划化、项目化、路径化。健全督办检查机制，对重点工作实行台帐管理、限时办结，认真落实月通报、季讲评和媒体公示制度。完善激励约束机制，继续实行干部即时记功，对不作为、慢作为、乱作为的干部实行严格问责，形成查处有力的长效问责机制。

　　（四）坚持依法行政。各级政府及其工作部门必须把依法行政贯穿于工作的全过程。创新行政执法监督机制，完善行政执法电子监察系统。严格执法程序，正确行使行政处罚自由裁量权。推进市、县两级行政复议委员会试点工作，努力打造全国行政复议示范城市。严格落实行政执法责任制和执法责任追究制，加大依法行政工作考核力度。自觉接受市人大和市政协的监督；加强电子政务平台建设，深化政务公开，将“市民问政”常态化，强化舆论监督和社会监督。

　　（五）加强作风建设。各级政府和部门领导干部必须严格执行中央、省委、市委关于改进工作作风的规定，向作风转变要推动工作的“正能量”。始终坚持以人为本、执政为民，规范和减少事务性活动，轻车简从深入基层调查研究、解决具体问题，始终保持同人民群众的血肉联系。认真落实党风廉政建设责任制，带头遵守廉政准则，时时处处严于律己、以身作则，廉洁从政、干净干事；切实抓好本地区本部门的廉政建设，坚决查处违法违纪案件，坚决纠正损害群众利益的各种不正之风。厉行勤俭节约，严格控制出国（境）访问，简化接待工作，严格控制接待和会议经费支出。建立健全行政权力运行制约和监督体系，完善公共资源配置、交易和生产管理制度，坚持用制度管权管事管人，让权力在阳光下运行。

　　各位代表！回顾过去一年，我们在困难中奋力前行，成绩来之不易；展望新的一年，我们在憧憬中肩负重任，更需加倍努力。让我们在市委的坚强领导下，脚踏实地、埋头苦干，夙夜在公、勤勉工作，为实现人民群众对美好生活的向往而努力奋斗！
